# Engineered bacteriophage lysins as novel anti-infectives

**DOI:** 10.3389/fmicb.2014.00542

**Published:** 2014-10-16

**Authors:** Hang Yang, Junping Yu, Hongping Wei

**Affiliations:** Key Laboratory of Special Pathogens and Biosafety, Center for Emerging Infectious Diseases, Wuhan Institute of Virology, Chinese Academy of SciencesWuhan, China

**Keywords:** lysin, chimeolysin, artilysin, bacteriophage, lysin engineering, enzybiotics

## Abstract

Bacteriophage lysins, the highly evolved specific peptidoglycan hydrolases, have long been demonstrated to be effective enzybiotics in various infectious models. The modular structure of lysins makes it possible to design bioengineered lysins that have desired properties, such as higher activity, or broader killing spectrum. Moreover, lysins can even be engineered to kill Gram-negative bacterial pathogens from without, a property that is not present in natural lysins. In this era of ever increasing multidrug resistant pathogens, engineered lysins represent a new class of enzybiotics that are powerful and readily available to fight antimicrobial resistance.

## Introduction

Bacteriophage lysins are novel murein hydrolases encoded by dsDNA phages in the late phase of infection cycle for the release of progeny virions (Loessner, 2005). These enzymes are usually genus-specific and highly active against bacterial peptidoglycan and capable of digesting the cell walls of susceptible bacteria, including multidrug resistant Gram-positive pathogens (Fischetti, 2006). Therefore, lysins have been considered as promising anti-infective enzybiotics with potential important applications in medicine and biotechnology in the current age of mounting antimicrobial resistance (Fischetti et al., 2006).

Currently, there are excellent reviews that have dealt with the characteristics of lysins and their applications, both as alternative enzybiotics in medical-oriented *in vitro* and *in vivo* tests (Fischetti, 2005; Fenton et al., 2010b; Knoll and Mylonakis, 2014), and as preservatives in the food industry (Callewaert et al., 2011; Oliveira et al., 2012). In the experimental models of sepsis (Schuch et al., 2002; Gilmer et al., 2013), pneumonia (Loeffler et al., 2001; Witzenrath et al., 2009), endocarditis (Entenza et al., 2005), meningitis (Grandgirard et al., 2008), nasopharyngeal infection (Kiser et al., 1999), canine pyoderma (Junjappa et al., 2013), endophthalmitis (Singh et al., 2014), skin and vaginal decolonization (Cheng et al., 2005), lysins have been used as effective anti-infectives to eliminate pathogens systemically and topically from mucosal surfaces and biofilms, including methicillin- and vancomycin-resistant *Staphylococcus aureus*, vancomycin-resistant *Enterococcus faecalis* and *E. faecium*, and penicillin-resistant *Streptococcus pneumoniae*. According to the opinion of Vincent A. Fischetti, several unique characteristics of lysins makes them attractive enzybiotics over small molecule antibiotics (Fischetti, 2008, 2010). These include (i) their specificity for the pathogens without disturbing the normal microflora, (ii) the low chance of developing bacterial resistance, and (iii) their ability to kill colonizing pathogens on mucosal surfaces. One possible problem of lysins may be their immunogenicity as protein molecules. However, studies thus far have illustrated that lysin-specific antibodies are non-neutralizing both *in vitro* and *in vivo* (Rashel et al., 2007; Daniel et al., 2010; Yang et al., 2014), which means that lysins can be used repeatedly in the treatment of infections caused by susceptible pathogens. Clinical trials are being conducted or prepared to assess the safety and pharmacokinetic properties of lysins in humans (Pastagia et al., 2013). For instance, a lysin against staphylococci P128 (George et al., 2012), has already stepped into phase II clinical trial (http://www.clinicaltrials.gov/ct2/show/NCT01746654?term=gangagen&rank=1) for studying its effectiveness in reducing the nasal carriage of *S. aureus* in humans. Although there are no lysins being used as medicine yet, it is believed that the breakthrough might come first for treating mucosal infections, such as nasal decolonization and wound healing. Furthermore, the combination of lysins with current antibiotics would be very effective to treat infections caused by multidrug resistant bacteria (Schuch et al., 2014).

Apart from natural lysins, there is an ever-growing interest in the engineered lysins created through modification or rational design from natural lysins. A perfect lysin that is ideal for anti-infective applications should maintain high bioavailability and activity. However, the genus-specific natural lysins are limited when treating infections caused by mixed bacteria from multiple genera. For example, a lysin with a relatively broad lytic spectrum that could lyse more than one genus of pathogens is preferred when treating mucous associated infections (human mucous membranes are the reservoir of many pathogenic bacteria including pneumococci, staphylococci and streptococci) (Coello et al., 1994; De Lencastre et al., 1999; Fischetti, 2003). Lysin engineering is of special promise to create enzybiotics with novel characteristics.

Chimeric lysins (also called chimeolysins) have been created by shuffling the domains, i.e., the cell wall binding domains (CBDs) and the catalytic domains (CDs) from natural lysins. Interestingly, the natural lysin Pal, identified from pneumococcal phage Dp-1, has proven to be a natural chimeolysin of intergeneric origin (Sheehan et al., 1997). Artificial lysins (also known as artilysins) have been created by fusing a natural lysin or part of its domain with another component that came from either a peptide or a protein. While natural lysins essentially lyse only Gram-positive bacteria exogenously, some artilysins could kill Gram-negative bacteria directly from without (Lukacik et al., 2012; Briers et al., 2014a). Therefore, developing engineered lysins may help to create novel enzybiotics with improved lytic activity and spectrum against both Gram-positive and negative bacterial pathogens, and provide novel clues to understand the modular evolution of lysins (Diaz et al., 1990). This review will outline the characteristics and the remarkable potency of engineered lysins in killing pathogenic bacteria both *in vitro* and *in vivo*.

## The action model of lysins

Differing from the traditional antibiotics, the anti-infective activity of lysin comes from its direct cell lysis upon contact with the bacterial cell wall. Lysins are expressed and accumulated in the cytosol of the host cell at the end of the phage replicative cycle (Young, 1992). With the help of another protein, the holin, lysins get access to their peptidoglycan substrate and cause rapid cell lysis (Wang et al., 2000). The holin-lysin system is essential for host cell lysis, and the molecular mechanisms underlying the procedure of “lysis from within” has been well discussed previously (Young and Wang, 2006).

When applied exogenously as recombinant enzymes, lysins have been demonstrated to cause rapid lysis of Gram-positive bacteria (Loessner et al., 1995). It is this potent ability to cause “lysis from without” of pathogenic Gram-positive cells upon direct contact with peptidoglycan that has laid the foundation of exploiting lysins as enzybiotics (Abedon, 2011). However, in the case of Gram-negative bacteria, the outer membrane hinders the access of lysins to their peptidoglycan substrates in the cell wall and therefore, their antibacterial is limited.

## The modular structure of lysin

**Figure 1:**
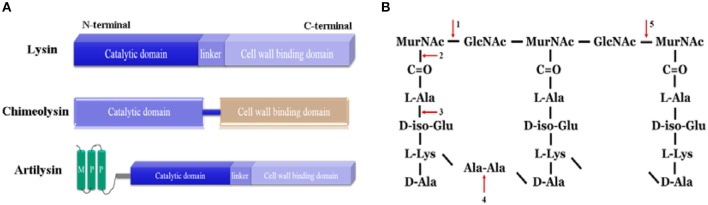
**Lysin-based murein hydrolases**. **(A)** The schematic structure of lysin, chimeolysin and artilysin. MPP, membrane penetrating peptides. **(B)** The cleavage sites of lysin-based murein hydrolases in the peptidoglycan. 1, N-acetyl muramidases; 2, N-acetylmuramoyl-L-alanine amidases; 3, L-alanoyl-D-glutamate endopeptidases; 4, interpeptide bridge endopeptidases; 5, N-acetyl-β-D-glucosaminidases.

Most frequently, lysins displayed a typically modular structure of at least two distinct domains (Villa and Crespo, 2010). That is an N-terminal CD and a C-terminal CBD, corresponding to their two basic functions: enzymatic hydrolysis and substrate recognition (Figure 1). In a few cases, lysins, particularly staphylococcal lysins, have been found to have more than one CDs and one CBD (Navarre et al., 1999; Rigden et al., 2003; Donovan et al., 2006b; Sass and Bierbaum, 2007; Obeso et al., 2008). Extraordinarily, a bacillus phage lysin, plyG, has been shown to have one CD and two separate binding domains, a CBD and a spore binding domain (SBD) (Yang et al., 2012). And the C1 streptococcal phage lysin, PlyC, is shown by crystallization to be a multimeric enzyme composed of eight cell wall binding subunits for each catalytic subunit (Nelson et al., 2006; McGowan et al., 2012).

Although CBD is necessary for most lysins, truncation of some lysins to remove the CBD can result in improved lytic activity without loss of specificity (Loessner et al., 1999; Horgan et al., 2009; Fenton et al., 2010a). One good example is lysin Ply187, the CD from lysin Ply187 (N-terminal 157 amino acids) has a much higher amidase activity than the whole lysin (Loessner et al., 1999).

Generally, the CD gets access to and specifically cleaves the major bonds in the peptidoglycan *via* the specific recognition of the CBD. The substrates of CBDs are postulated to be unique and conserved molecules in the cell walls that are essential for bacterial viability, usually neutral polysaccharides that are restricted to particular species or even strains. For instance, pneumococcal phage lysin targets choline, an indispensable cell wall molecule for anchoring in *S. pneumonia* (Garcia et al., 1988; Hermoso et al., 2003). The CBDs of *Listeria* phage lysins can even distinguish various serotypes of *Listeria* species in liquid or food samples (Loessner et al., 2002; Schmelcher et al., 2010; Eugster et al., 2011; Eugster and Loessner, 2012). Because of the relatively independent functions of these two domains, engineered lysins can be constructed by shuffling these domains from different origin or fusing them with other molecules. By doing so, the chimera may be empowered with new characteristics, including binding specificity, killing spectrum, solubility, stability, activity and so on.

## Chimeolysin

A chimeolysin could be designed to have an improved lytic activity, or a broader lytic spectrum, compared with its parental enzyme (Table 1). For instance, the specific lytic activity of Ply187AN-KSH3b, a chimeolysin constructed by fusing the amidase of lysin Ply187 with the LysK SH-3b CBD, was 10-fold higher than that of its parental lysin Ply187AN (Mao et al., 2013). Specifically, by adding the non-SH3b-like CBD of phiNM3 to the CD of Ply187 (giving rises to chimeolysin ClyH) yielded a 3.7–13.6 fold increase in lytic activity against *S. aureus* (Yang et al., 2014). ClyH could even lysis *S. sobrinus*, a streptococci that Ply187 could not lyse at all. Similar results are seen with a chimeolysin Ply187N-V12C, however in this case, the chimeolysin has an extended lytic activity against *S. dysgalactiae*, the main pathogen of cow mastitis (Dong et al., 2014).

**Table 1:** **The structural composition and characteristics of engineered lysins**.

	**N-terminal donor**	**C-terminal donor**	**Property**	**Antimicrobial spectrum**	**Ref**.
**CHIMEOLYSIN**					
ClyS	Phage Twort lysin plyTW, endopeptidase	phiNM3 lysin	Highly soluble, superiority to mupirocin for skin decolonization	Staphylococci	Daniel et al., 2010; Pastagia et al., 2011
Lys168-87	*E. faecalis* phage F168/08 lysin, CHAP^a^	Phage 87 lysin Lys87^b^	Highly soluble, broad antimicrobial activity	Staphylococci, *E. faecalis, E. faecium, S. pyogenes*	Fernandes et al., 2012
Lys170-87	*E. faecalis* phage F170/08 lysin, amidase	Phage 87 lysin Lys87^b^	Highly soluble, broad antimicrobial activity	Staphylococci, *E. faecalis, E. faecium, S. pyogenes*	Fernandes et al., 2012
PRF-119	Phage K lysin plyK, CHAP	Lysostaphin, SH3b like domain	Very good activity	Staphylococci	Idelevich et al., 2011
λ SA2-E-Lyso-SH3b	Phage λ SA2 lysin, endopeptidase	Lysostaphin, SH3b like domain	Increased activity and extended lytic spectrum	Staphylococci, Streptococci	Becker et al., 2009; Schmelcher et al., 2012
λ SA2-E-LysK-SH3b	Phage λ SA2 lysin, endopeptidase	LysK, SH3b like domain	Increased activity and extended lytic spectrum	Staphylococci, Streptococci	Becker et al., 2009; Schmelcher et al., 2012
B30-182-Lyso	Phage B30 lysin, endopeptidase	Lysostaphin, SH3b like domain	Extended lytic spectrum and binding capacity	*S. aureus, S. uber, S. agalactiae, S. dysgalactiae*	Donovan et al., 2006a
Ply187AN-KSH3b	Phage 187 lysin Ply187, amidase	LysK, SH3b like domain	Improved lytic activity	Staphylococci	Mao et al., 2013
ClyH	Phage 187 lysin Ply187, amidase	phiNM3 lysin	Improved lytic activity and extended lytic spectrum	Staphylococci, S. *sobrinus*	Yang et al., 2014
Ply187N-V12C	Phage 187 lysin Ply187, amidase	Lysin PlyV12, SH3b like domain	Extend lytic spectrum	Staphylococci, *Streptococci, Enterococci*	Dong et al., 2014
**ARTILYSIN**					
P128	tail-associated enzyme of Phage K	Lysostaphin, SH3b like domain	High lytic activity	Staphylococci	Vipra et al., 2012
P16-17	Phage p68 lysin p16, CHAP	Minor coat protein 17 of phage p68^b^	Highly soluble	*S. aureus*	Manoharadas et al., 2009
CLL	lysin Cpl-1, lysozyme	LytA C-terminal domain	Altered binding capacity	Streptococci	Diaz et al., 1990
CLA	LytA amidase domain	Lysin Cpl-1 binding domain	Altered binding capacity	Streptococci	Diaz et al., 1990
Art-085	SMAP-29 peptide	Lysin KZ144	Kills Gram-negative bacteria	*P. aeruginosa, P. syringae, P. putida*	Briers et al., 2014a
Pesticin-like	T4 lysozyme	Pesticin	Kills Gram-negative bacteria	Yersinia, *E. coli* expressing FyuA	Lukacik et al., 2012
LoGT series	Various peptides^c^	Various lysins^d^	Kills Gram-negative bacteria	*P. aeruginosa, A. baumannii, E. coli, S. Typhimurium*	Briers et al., 2014b

a *CHAP: cysteine and histidine-dependent aminopeptidase/hydrolase*.

b *The characters of these CBDs cannot be confirmed for their sequences are unavailable*.

c *These peptides include α4, MW1, MW2, polycationic peptide (PCNP), hydrophobic pentapeptide (HPP), Parasin1 (Pa1), and lycotoxin1 (Ly1)*.

d *These lysins include OBPgp279 (YP_004958186.1), PVP-SE1gp146 (YP_004893953.1), phiKZgp144 (NP_803710.1), 201ϕ 2-1gp229 (YP_001956952.1), CR8gp3.5, P2gp09 (NP_046765.1), and PsP3gp10 (NP_958065.1)*.

In some cases, the lytic spectrum of a chimeolysin may be altered by changing its CBD. The streptococcal λ Sa2 prophage endolysin has a strong lytic activity against multiple streptococcal strains but not staphylococcal pathogens (Donovan and Foster-Frey, 2008). Replacing its Cpl-7 CBD with a staphylococcal SH3b domain from either lysostaphin or LysK resulted in a 5-fold increase in staphylolytic activity, and surprisingly, the chimera also maintained significant streptolytic activity (Becker et al., 2009). Similarly, bioengineering different CBDs of Listeria phage lysins provides extended recognition and binding properties (Schmelcher et al., 2011).

Daniel et al. constructed chimeolysins to solve the solubility problems associated with natural lysins. By fusing the CD of phage Twort lysin to the CBD of phiNM3 lysin, they created a highly soluble chimera, ClyS, which was shown to have potent anti-infective efficacy against MRSA in a murine sepsis model (Daniel et al., 2010). ClyS has also shown superior to mupirocin for skin decolonization of methicillin-resistant and -sensitive *S. aureus* strains in mice (Pastagia et al., 2011). In another work, Fernandes et al. were able to improve the solubility and antimicrobial activity of Lys87, *via* substituting its CD with a CD from an enterococcal lysin that is highly soluble (Fernandes et al., 2012).

There is also an example of a chimeolysin that was designed to avoid resistance to phages. The chimera PRF-119 has been proven to kill four phage-resistant *S. aureus* mutants (Idelevich et al., 2011), and at the same time, showed very good activities against *S. aureus* with MIC_90_ of 0.391 μg/ml for 398 MSSA and 776 MRSA clinical isolates, respectively.

## Artilysin

Artilysin denotes an engineered enzybiotic created by fusing a fragment of a natural lysin with peptides or other proteins (Figure 1). One outstanding application is to design artilysins against Gram-negative bacteria (Table 1). In recent research, Briers et al. (2014a) designed an artilysin with a highly efficient antibacterial activity against multidrug resistant strains and persisters of *Pseudomonas aeruginosa*, by fusing a sheep myeloid antimicrobial peptide with 29 amino acids residues (SMAP-29) to the N-terminus of the endolysin KZ144. SMAP-29 is an α-helical cathelicidin found in sheep leukocytes that can pass the outer membrane of Gram-negative bacteria via a self-promoted uptake pathway (Skerlavaj et al., 1999). It is well known that antimicrobial peptides kill bacteria either by disrupting the cytoplasmic membrane or by crossing the membrane and acting on intracellular targets, however, such peptides are cytotoxic to mammalian cells (Maher and McClean, 2006; Dawson and Liu, 2009). Meanwhile, the natural form of lysin KZ144 could not kill *P. aeruginosa* cells directly, but was effective against membrane-permeated cells, which are obtained by treatment with chloroform (Briers et al., 2007), or under high hydrostatic pressure (Briers et al., 2008). Surprisingly, the artilysin overcomes the disadvantages of its both donors. The artilysin not only kills the *P. aeruginosa* cells directly, but also shows no cytotoxicity, indicating that the application of bacteriophage lysins as enzybiotics must not be limited only to Gram-positive pathogens.

Very recently, Briers and coworkers described a series of artilysins that are highly active against Gram-negative pathogens. For instance, artilysin LoGT-23, can cause a reduction of 5.5 log, 5.2 log, 2.4 log and 1.5 log for *P. aeruginosa, Acinetobacter baumannii, E. coli* and *Salmonella Typhimurium* within 30 min in the presence of 0.5 mM EDTA, respectively. The *in vivo* efficacy of some artilysins have also been tested in a *Caenorhabditis elegans* bacterial infection model, indicating that artilysins may have broad anti-infective applications.

In another example, Lukacik et al. constructed a pesticin-like hybrid toxin that kills specific *Yersinia* and pathogenic *E. coli* strains, by attaching an FyuA targeting domain to the N-terminus of T4 lysozyme (Lukacik et al., 2012). The hybrid could pass the out membrane in order to reach the peptidoglycan layer through the interaction with the outer membrane transporter FyuA. Moreover, the hybrid toxin can evade the pesticin immunity protein (Pim) indicating that it may be a potential candidate for *in vivo* therapy over pesticin. Because FyuA is more common in pathogenic bacteria, the hybrid toxin harbors a great potential to kill pathogenic bacteria specifically.

## Concluding remarks

Bacteriophage lysins demonstrate several highly desirable properties compared with antibiotics, which include novel antimicrobial mechanisms, high specificity and activity against multidrug resistant pathogens, as well as a low possibility of developing resistance. The huge number of phages existing on earth supports great resources for lysin discovery. Meanwhile, the modular structure of lysins provides a great chance to create engineered lysins with desired properties, which may include extended killing spectra, enhanced killing activity, and improved solubility. Through rational design of natural lysins, artilysins can even kill Gram-negative bacteria that are resistant to natural lysins. Taken all these together, engineered lysins represent a new class of enzybiotics that are powerful and readily available to fight the emerging antimicrobial resistance.

### Conflict of interest statement

The authors declare that the research was conducted in the absence of any commercial or financial relationships that could be construed as a potential conflict of interest.
